# Infection by agnoprotein-negative mutants of polyomavirus JC and SV40 results in the release of virions that are mostly deficient in DNA content

**DOI:** 10.1186/1743-422X-8-255

**Published:** 2011-05-24

**Authors:** Ilker K Sariyer, Abdullah S Saribas, Martyn K White, Mahmut Safak

**Affiliations:** 1 Department of Neuroscience, Laboratory of Molecular Neurovirology, Temple University School of Medicine, 3500 N. Broad Street, Philadelphia PA 19140 USA

**Keywords:** JC virus, BK virus, SV40, replication, transcription, virion release

## Abstract

**Background:**

Human polyomavirus JC (JCV) is the etiologic agent of a brain disease, known as progressive multifocal leukoencephalopathy (PML). The JCV genome encodes a small multifunctional phospho-protein, agnoprotein, from the late coding region of the virus, whose regulatory functions in viral replication cycle remain elusive. In this work, the functional role of JCV and SV40 agnoproteins in virion release was investigated using a point mutant (Pt) of each virus, where the ATG codon of agnoprotein was mutated to abrogate its expression.

**Results:**

Analysis of both viral protein expression and replication using Pt mutant of each virus revealed that both processes were substantially down-regulated in the absence of agnoprotein compared to wild-type (WT) virus. Complementation studies in cells, which are constitutively expressing JCV agnoprotein and transfected with the JCV Pt mutant genome, showed an elevation in the level of viral DNA replication near to that observed for WT. Constitutive expression of large T antigen was found to be not sufficient to compensate the loss of agnoprotein for efficient replication of neither JCV nor SV40 in vivo. Examination of the viral release process for both JCV and SV40 Pt mutants showed that viral particles are efficiently released from the infected cells in the absence of agnoprotein but were found to be mostly deficient in viral DNA content.

**Conclusions:**

The results of this study provide evidence that agnoprotein plays an important role in the polyomavirus JC and SV40 life cycle. Infection by agnoprotein-negative mutants of both viruses results in the release of virions that are mostly deficient in DNA content.

## Background

A large number of studies indicate that the small regulatory proteins of many viruses play important roles in various aspects of viral infection cycle, including replication [1-3], transcription [4-10], translation [11], export of viral transcripts from nucleus to cytoplasm [12], viral assembly [13] and release of viral particles [14,15]. In addition, these proteins may also modulate host-cell functions by deregulating the expression of key cellular genes [16]. Therefore, such regulatory proteins are important for successful completion of the viral life cycle and study of their regulatory roles in viral life cycle is critically important for understanding of the viral replication process and the disease progression that respective viruses induce in their host.

The late coding region of human polyomavirus JC (JCV) and simian virus 40 (SV40) encodes a small regulatory phosphoprotein, agnoprotein, whose expression during the viral lytic cycle has been demonstrated by biochemical and immunocytochemical methods [17-19]. Agnoprotein is a cytoplasmic protein predominantly localized to the perinuclear region of infected cells. A small amount of agnoprotein is also detected in nucleus in the infected cells. The expression pattern of agnoprotein in tissue sections from progressive multifocal leukoencephalopathy (PML) has also been analyzed and also shown to localize to the cytoplasmic and perinuclear regions of the infected brain cells from PML patients [20]. Amino acid sequence alignment of the agnoproteins for JCV, BKV and SV40 shows a high degree of sequence identity of about 70% [10,21]. While the amino-terminal and central regions of each agnoprotein exhibit considerable sequence identity with one another, sequences toward the carboxy-terminal region are more divergent.

JCV is the etiologic agent of the fatal demyelinating disease of the brain, PML [7,22-25] and its late gene product, agnoprotein, has been previously shown to functionally interact with other JCV regulatory proteins, including large T-antigen [10] and small t-antigen [26] and several cellular factors [16,19]. In addition, agnoprotein has been shown to have inhibitory effects on cell cycle progression [16]. Mutational analysis of agnoprotein from the closely related virus SV40 suggested that it may have effects on various aspects of the viral lytic cycle including transcription, translation, virion production and maturation of the viral particles [27-34]. It has been known for more than a decade that SV40 and BKV agnoproteins are phosphorylated but no function has yet been assigned to this modification [18,35]. More recent studies explored the possibility that potential phosphorylation sites of agnoprotein are the targets for well-characterized protein kinases, including protein kinase C (PKC). Indeed, these studies demonstrated that agnoprotein is phosphorylated by PKC and phosphorylation turns out to play a significant role in the function of this protein during the viral replication cycle [36,37]. More recent reports also showed that agnoprotein deletion mutants are non-functional but can be rescued by trans-complementation [36,38]. In addition, it has been suggested that agnoprotein aids in the release of virions from infected cells [39].

In order to delineate whether agnoprotein is involved in release of viral particles from infected cells, we have utilized point mutants of JCV and SV40 agnoproteins in which the ATG translation initiation codon of agnogene was altered and thereby the expression of the protein was ablated. In this report, we provide experimental evidence indicating that both JCV and SV40 virions are efficiently released from the infected cells in the absence of agnoprotein, however, the released viral particles are mostly deficient in DNA content, which greatly hampers the ability of the propagation of the mutant virus relative to wild-type.

## Methods

### Cell lines

Primary human fetal glial (PHFG) cells were prepared as follows: Brain tissue from an aborted 16-week-old fetus was first cut into small pieces in Hanks balanced salt solution (HBSS) and the clumps were mechanically disrupted by repeated pipetting of the soft tissue. To further separate the cells, the tissue was incubated at 37°C with trypsin (0.005%) and DNAse I (50 μg/ml) for 30 min. Fetal bovine serum (0.1%) was then added to inactivate trypsin and the cells were washed with HBSS twice to remove trypsin. The cells were then passed through a 70 mm mesh to remove larger cell clumps. The cells in the filtrate were spun down and the pellet was resuspended in a small volume of HBSS with a fire polished glass pipette to obtain single cells. After cell viability and count were determined, cells were resuspended in growth media D-MEM+F12 containing fetal bovine serum, FBS (10%), L-glutamine (2 mM), insulin (2.5 mg/l) and gentamycin (50 mg/l). The cells were plated on collagen-coated flasks at a concentration of 2-10 × 10^6 ^per 75 cm^2 ^flask and incubated at 37°C. SVG-A is a subclonal population of a human glial cell line which was established by transformation of human fetal glial cell line with an origin-defective SV40 mutant [40]. SVG-A cells were grown in Dulbecco's Modified Eagle's Medium (DMEM) supplemented with 10% heat-inactivated fetal bovine serum (FBS) and antibiotics (penicillin/streptomycin, 100 μg/ml). They were maintained at 37°C in a humidified atmosphere with 7% CO_2. _Cos-7 cells were grown in Dulbecco's Modified Eagle's Medium (DMEM) supplemented with 10% (FBS) and antibiotics (penicillin/streptomycin, 100 μg/ml). They were maintained at 37°C in a humidified atmosphere with 7% CO_2._

### Stable cell lines

SVG-A cells (2 × 10^5 ^cells/100 mm tissue culture dish) were either stably transfected with pcDNA3-JCV agnoprotein expression plasmid (pcDNA_3_-JCV-Agno) (5 μg/plate) [10] or pcDNA_3 _vector alone (Invitrogen) (5 μg/plate) by lipofectin method according to manufacturer's recommendations (Invitrogen). At five hour posttransfection, transfectants were washed twice with phosphate buffered saline (PBS) and refed with DMEM containing 10% FBS. After twenty-four hours, cells from each 100-mm plate were trysinized and replated onto ten 100-mm plates. Cells were allowed to attach for six hours and then the medium was replaced with DMEM containing 400 μg/ml G418 and 10% FBS. The medium was changed every three days until individual colonies formed. Individual colonies were randomly selected, screened for agnoprotein expression by Western blotting, expanded and frozen in liquid nitrogen.

### Plasmid constructs

Description of both JCV WT (Bluescript KS+JCV-Mad-1 WT) and JCV agnoprotein point mutant (Bluescript KS+JCV-Mad-1 Pt) was previously described [36]. Simian virus 40 genome [SV40(776)] was subcloned from pBR322 vector into Bluescript KS+ at the *Bam*H I site and designated as Bluescript KS+SV40(776) WT. SV40 agnoprotein Pt mutant at ATG site was created by an "Excite™ PCR-based site-directed mutagenesis Kit" (Stratagene) utilizing the following primers: Primer 1. SV40(776) (331-362): 5'-GG AGA TCT TGC TGC GCC GGC TGT CAC GCC AGG-3'. Primer 2. SV40(776) (330-309): 5'-TGA AAT AAC CTC TGA AAG AGG-3'. Underlined sequence represents the substitution of *Nco *I site with a newly created *Bgl *II site. PCR was performed according to manufacturer's recommendations. Briefly, 10 ngs of Bluescript KS+SV40(776) WT plasmid were PCR amplified with 15 picomols of each primer shown above under stringent reaction conditions. Primers were phosphorylated by T4 polynucleotide kinase at 5'-ends before PCR reaction. After initial denaturation of the plasmid for 10 min at 94°C, reaction was cycled 12 times under the following parameters: 95°C, 2 min; 56°C, 2 min; and 72°C 8 min; and 72°C 10 min for one cycle. Upon PCR reaction, the PCR product was treated with *Dpn *I enzyme to eliminate template DNA, religated and transformed into supercompetent Epicurian *Coli *XL-1-Blue supercompetent cells (Stratagene). In the point mutant construct, *Nco *I site (CCATGG) of WT strain containing the translation initiation codon (ATG) of agnoprotein was converted into *Bgl *II site (AGATCT) by base substitution which allowed us to distinguish the mutant from WT by restriction enzyme digestion. Finally, base substitutions and overall integrity of the viral DNA sequences were verified by DNA sequencing for SV40 Pt and JCV Pt plasmids. pcDNA_3_-JCV-Agnoprotein expression plasmid (pcDNA_3_-JCV-Agno) was described previously [10].

### Transfections

Both PHFG and SVG-A cells (2 × 10^6 ^cell/75 cm^2 ^flask) were transfected either with Mad-1 WT (8 μg) or Mad-1 Pt (8 μg) mutant JCV viral DNA using lipofectin-2000 according to manufacturer's recommendations (Invitrogen). CV-1 cells (1.5 × 10^6 ^cell/75 cm^2 ^flask) were transfected either with SV40(776) WT (8 μg) or SV40(776) Pt (8 μg) mutant viral DNA using lipofectin-2000. At five hour posttransfection, cells were washed twice with PBS. Cos-7 cells were transfected as described for CV-1 cell lines. PHFG cells were fed with D-MEM+F12 containing FBS (10%), L-glutamine (2 mM), insulin (2.5 mg/l) and gentamycin (50 mg/l); and SVG-A, Cos-7 and CV-1 cells were fed with DMEM supplemented with 5% FBS.

### Western blotting

Whole-cell extracts prepared from PHFG cells untransfected or transfected with either WT JCV Mad-1 genome or JCV Mad-1 Pt mutant genome at 7d, 14d, and 21d posttransfection were resolved on SDS-15% PAGE and blotted onto nitrocellulose membranes. Blotted membranes were probed with an anti-agnoprotein polyclonal antibody as described previously [19] and detected by a chemiluminescence (ECL) method according to the manufacturer's recommendations (Amersham). In parallel, nuclear extracts prepared from either transfected or untransfected cells were analyzed for viral VP1 and large T antigen (LT-Ag) by Western blotting utilizing a monoclonal anti-VP1 antibody (PAb 597) (a kind gift from Dr. W. Atwood, Brown University, Rhode Island) and a monoclonal anti-SV40 LT-Ag antibody (Ab-2), which is cross-reactive with JCV LT-Ag respectively. Similarly, whole-cell and nuclear extracts prepared from SVG-A cells stably expressing JCV agnoprotein which were either transfected with JCV Mad-1 WT or JCV Mad-1 Pt mutant were analyzed for agnoprotein. In addition, whole-cell and nuclear extracts were prepared from CV-1 cells untransfected or transfected with either SV40(776) WT genome or SV40(776) Pt mutant genome at indicated time points posttransfection. Extracts were then analyzed for SV40 agnoprotein, LT-Ag and VP1 expression by Western blotting using anti JCV agnoprotein, anti-SV40 LT-Ag (Ab-2) and anti-JCV VP1 (PAb597) antibodies. Western blots were probed with an anti-lamin A antibody to demonstrate the equal loading of the protein extracts.

### Replication assay

Replication assays were carried out as previously described [10]. Briefly, PHFG cells or SVG-A cells (2 × 10^6 ^cells) grown in 75 cm^2 ^flasks were transfected either with JCV Mad-1 WT or JCV Mad-1 Pt mutant viral genome (8 μg/2 × 10^6 ^cells/75 cm^2 ^flask) by lipofectin-2000 transfection method. Of note, The pBluescript (back bone) vector from both JCV and SV40 plasmids was digested with *Bam*H I before using the plasmids in transfections. Lipofectin-DNA mixture was incubated with cells for 5 h and washed with PBS. Transfected SVG-A cells were fed with complete D-MEM media with 3% FBS. Transfected PHFG cells were fed with a special growth media D-MEM+F12 media containing 10% FBS, L-Glutamine (2 mM), gentamycin (50 mg/l) and insulin (2.5 mg/l). At indicated time points posttransfection, low-molecular-weight DNA containing both input and replicated viral DNA was isolated by the Hirt method [41], digested with *Bam*H I and *Dpn *I enzymes, resolved on a 1% agarose gel and analyzed by Southern blotting.

### Indirect immunofluorescence microscopy

Indirect immunofluorescence microscopy studies were performed as previously described [19,42,43]. Briefly, CV-1 cells transfected with either SV40(776) WT or its Pt mutant genome were seeded at subconfluency on polylysine-coated glass chamber slides at 5^th ^day posttransfection. The next day, cells were washed twice with PBS and fixed in cold acetone. Fixed cells were blocked with 5% bovine serum albumin in PBS for 2 h and incubated with a monoclonal primary anti-SV40 large T antigen (Ab-2) antibody overnight. Cells were subsequently washed four times with PBS-0.01% Tween 20 for 10-min intervals and incubated with an anti-mouse fluorescein isothiocyanate (FITC)-conjugated goat anti-mouse secondary antibody for 45 min. Finally, the slides were washed three times with PBS, mounted, and examined under fluorescence microscope for detection of LT-Ag. Indirect immunofluorescence microscopy studies were performed for JCV Mad-1 WT and its Pt mutant. SVG-A cells transfected with either JCV Mad-1 WT or its Pt mutant genome were seeded at subconfluency on polylysine-coated glass chamber slides at 6^th ^day posttransfection and processed for detection of agnoprotein and VP1 as described above for SV40 using anti-Agno polyclonal [19] and anti-VP1 monoclonal (PAb597) primary antibodies followed by incubation with a FITC-conjugated goat anti-rabbit and Rhodamine-conjugated goat anti-mouse secondary antibodies respectively as described in each figure legend. Indirect immunofluorescence microscopy studies were also performed for SV40(776) WT and its Pt mutant in Cos-7 cells as described in the respective figure legend.

### Viral particle release assay

Mad-1 WT genome or its agnoprotein Pt mutant were separately transfected into SVG-A cells (8 μg DNA/2 × 10^6 ^cells/75 cm^2 ^flask). Supernatants from infected cells were collected at indicated time points, centrifuged at 16,000×g to clear cell debris and were subjected to immunoprecipitation using an anti-VP1 antibody (PAb597) (2 μg). Half of the samples were analyzed by Western blotting using anti-VP1 antibody (PAb597), the other half was analyzed by Southern blotting for detection of encapsidated viral DNA. The viral DNA from capsids was purified employing Qiagen spin columns [44], digested with *Bam*H I and *Dpn *I enzymes, resolved on an 1% agarose gel and analyzed by Southern blotting using probes prepared from whole Mad-1 genome. In parallel, SV40(776) WT or its Pt mutant DNA was transfected into CV-1 cells (8 μg/2 × 10^6 ^cells/75 cm^2 ^flask) and supernatants of the samples were collected at indicated time points and processed as described above for JCV Mad-1 WT and its Pt mutant.

## Results

### Both viral early and late protein expression are markedly reduced in the absence of agnoprotein

**Figure 1:**
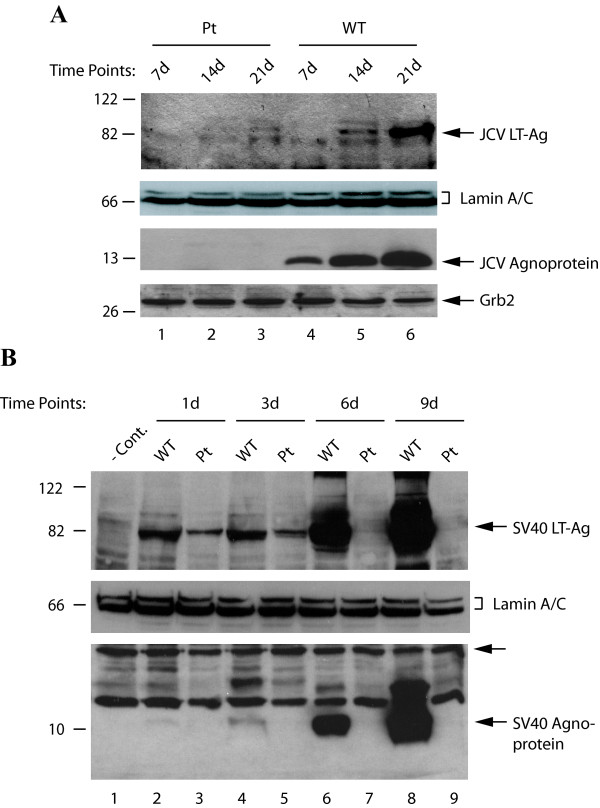
**Analysis of the expression levels of LT-Ag from JCV Pt and SV40 Pt backgrounds**. (A) PHFG cells were transfected/infected either with JCV Mad-1 WT or its Pt mutant genome, nuclear and whole-cell extracts prepared at the indicated time points and analyzed by Western blot for LT-Ag and agnoprotein respectively. Lamin A/C was also probed for equal loading of the nuclear extracts for LT-Ag using anti-lamin A/C polyclonal antibody (Cat #: 2032, Cell signaling). Western blotting with anti-Grb2 monoclonal antibody (Santa Cruz, C-7, Cat #: sc-8034) demonstrates the equal loading of the samples for agnoprotein expression (bottom panel). (B) CV-1 cells were transfected either with SV40(776) WT or its Pt mutant genome, the whole-cell and nuclear extracts prepared at the days indicated and analyzed by Western blot for agnoprotein and LT-Ag expression. An unlabeled arrow points to the band that is recognized by anti-Agno antibody which serves as a loading control for the agnoprotein samples. In lane 1, nuclear extracts prepared from untransfected cells were loaded as negative control (- Cont.).

JCV agnoprotein is a small basic phospho-protein and little is known about its regulatory roles in viral replication cycle. In this study, we investigated whether indeed JCV and SV40 agnoproteins play regulatory roles in release of virions from infected cells, utilizing agnoprotein point mutants of each virus. We first sought to determine whether JCV agnoprotein plays a role in regulation of the viral early gene expression by introducing JCV Mad-1 WT and its Pt mutant genomes into PHFG by transfection and analyzing the protein expression by Western blotting. At 7d, 14d and 21d after transfection, nuclear extracts were prepared and analyzed to determine the level of LT-Ag expression by immunoblotting. Of note, the virus infection cycle initiates upon transfection of the viral genome into the permissive cells. As shown in Figure 1A, the level of LT-Ag expression from the WT early promoter gradually increased as the JCV infection cycle reached 21d posttransfection (lanes 4-6). In contrast, the level of expression of LT-Ag was almost undetectable in nuclear extracts prepared from the cells transfected with its Pt mutant genome at days 7 and 14 (lanes 1 and 2), and barely detectable for 21d posttranfection (lane 3). Comparison of the band intensities corresponding to LT-Ag for WT versus Pt mutant genomes at 21d posttransfection by densitometry showed that JCV early gene expression was reduced ~25 fold in the absence of agnoprotein (lanes 3 and 6 respectively). In parallel, absence and presence of the agnoprotein expression in whole-cell extracts was analyzed by immunoblotting from the JCV Pt mutant and the WT backgrounds (Figure 1A bottom panel). While expression of agnoprotein from the WT background was readily detectable (lanes 4-6), as expected, its expression was absent from the Pt background (lanes 1-3).

In the case of SV40, we also assessed the effect of agnoprotein on viral early gene expression using its Pt mutant genome. CV-1 cells, which are permissive for SV40 replication, were transfected with either SV40(776) WT or its Pt mutant genome. Nuclear extracts were prepared at the time points indicated and analyzed by Western blotting for the expression of SV40 LT-Ag. As shown in Figure 1B, expression of LT-Ag from both WT and the mutant backgrounds 24h (1d) after transfection (upper panel, lane 2) was readily detectable but the level of SV40 LT-Ag expression in cells transfected with the Pt mutant genome decreased significantly (compare lane 3 with 2). A similar expression profile was observed for both mutant and WT viruses even at 3d after transfection. Interestingly, at 6d and 9d after transfection, whereas the level of LT-Ag expression from WT background gradually but substantially increased (lanes 6 and 8), that of LT-Ag from the Pt mutant background reduced to barely detectable levels (lanes 7 and 9). In parallel, we analyzed the presence and absence of agnoprotein expression in whole cell extracts from SV40 WT and SV40 Pt mutant backgrounds respectively (Figure 1B, bottom panel). While the expression of SV40 agnoprotein was readily detectable in CV-1 cells transfected with WT genome (lanes 4, 6 and 8), as expected, its expression was not evident in cells transfected with SV40 Pt mutant genome (lanes 3, 5, 7 and 9).

**Figure 2:**
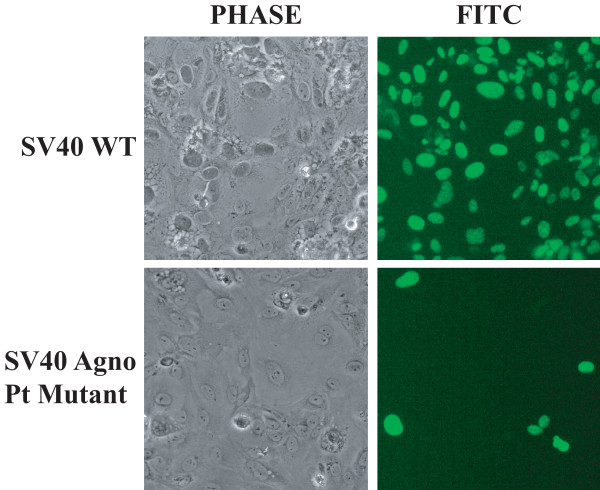
**Immunocytochemical analysis of CV-1 cells, transfected/infected either with SV40(776) WT or the Pt mutant genome, for detection of LT-Ag expression**. CV-1 cells were transfected either with SV40(776) WT or the Pt mutant genome, fixed at 4^th ^day posttransfection, incubated with a primary anti-SV40 LT-Ag (Ab-2) antibody followed by incubation with a FITC-conjugated goat anti-mouse secondary antibody and examined under a fluorescence microscope for detection of SV40 LT-Ag expression.

We next analyzed the level of SV40 early gene expression in the absence of agnoprotein by immunocytochemistry for LT-Ag. CV-1 cells were transfected with either WT or Pt SV40 mutant genome, fixed at 4d after transfection, incubated with a monoclonal antibody to SV40 LT-Ag (Ab-2) and then incubated with a FITC-conjugated secondary antibody. Examination of the cells under a fluorescence microscope for LT-Ag expression showed that SV40 WT infected close to 100% of the cells by day 4, (i.e., nearly all cells were immunocytochemically positive for LT-Ag) (Figure 2, upper panels). In contrast, SV40 Pt mutant was able to infect less than 1-5% of the cell population as assessed by detection of LT-Ag at day 4 after transfection (Figure 2, lower panels).

**Figure 3:**
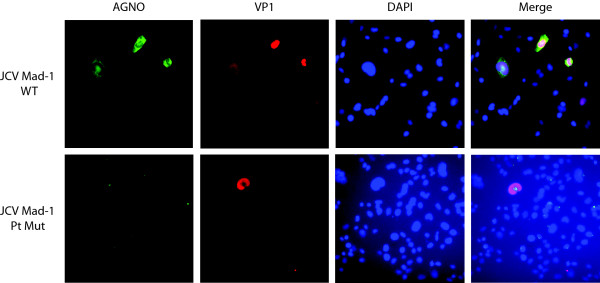
**Immunocytochemical analysis of SVG-A cells transfected/infected either with JCV Mad-1 WT or its Pt mutant genome for detection of agnoprotein (AGNO) and VP1 expression**. SVG-A cells were transfected either with JCV Mad-1 WT or the Pt mutant genome, fixed at 6^th ^day posttranfection, incubated with a primary anti-Agno polyclonal and anti-VP1 (PAb597) monoclonal antibodies followed by incubation with a FITC-conjugated goat anti-rabbit and Rhodamine-conjugated goat anti-mouse secondary antibodies and examined under a fluorescence microscope for detection of JCV agnoprotein and VP1 expression. DAPI staining was also performed to visualize the nucleus of the cells.

As analyzed for the expression level of SV40 early genes (Figure 2), we also analyzed the level of VP1 expression for JCV Mad-1 strain and its Pt mutant in the absence of agnoprotein in SVG-A cells by immunocytochemistry (Figure 3). Transfected/infected cells were fixed at 6^th ^day posttransfection and analyzed for the late capsid gene expression, VP1, as described in the figure legend. Since JCV is a slow growing virus compared to SV40 in tissue culture, a small number of cells were found to be positive for both JCV WT and its Pt mutant. However, our data showed that the number of positive cells for VP1 expression for Pt were always less than that of WT, which is consistent with our findings for the SV40 Pt mutant (Figure 2).

**Figure 4:**
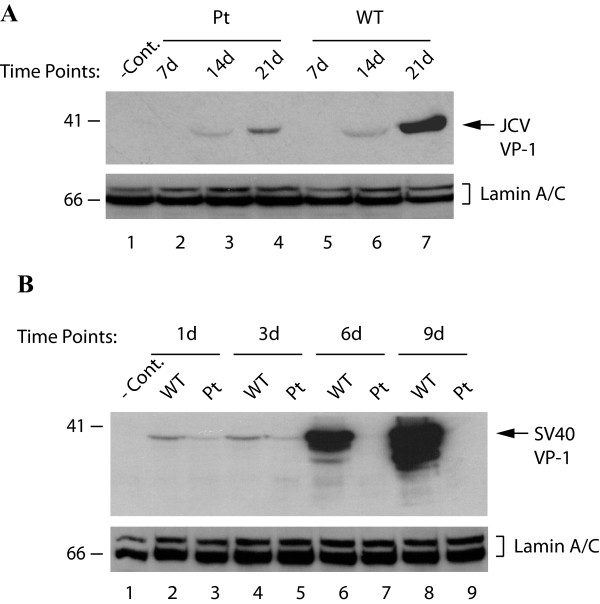
**Analysis of late capsid protein (VP1) expression in PHFG cells transfected/infected with either JCV Mad-1 WT or its Pt mutant genome**. (A) Nuclear extracts were prepared from PHFG cells transfected either with JCV Mad-1 WT or Pt mutant genome and analyzed by Western blot for VP1 expression. (B) CV-1 cells were transfected either with SV40(776) WT or the Pt mutant genome; and the nuclear extracts were prepared at the days indicated and analyzed by Western blot for VP1 expression. In lane 1 in each panel, nuclear extracts prepared from untransfected cells were loaded as negative control (- Cont.). Blots for panel A and B were also analyzed for Lamin A and C expression to demonstrate equal loading of nuclear proteins onto the gels.

We also compared the viral late gene expression from WT versus Pt backgrounds by Western blotting for VP1. Nuclear extracts were prepared at 7d, 14d and 21d after transfection of PHFG cells with JCV Mad-1 WT or Pt mutant and analyzed by Western blot using a monoclonal anti-JCV VP1 antibody. As shown in Figure 4A, although there was a gradual increase in the level of VP1 expression from both WT and the mutant backgrounds as the infection cycle progressed over the 21 day time course, we observed a significant reduction in the level of VP1 expression from the mutant compared to WT (compare lanes 4 and 7).

The effect of SV40(776) Pt mutant on viral late gene expression was also analyzed. Nuclear extracts prepared from CV-1 cells transfected with either SV40(776) WT or the Pt mutant genome were analyzed by Western blot for VP1. As shown in Figure 4B, the level of SV40 VP1 expression, although demonstrated a relatively low but steady expression for 1 and 3 days after transfection (lanes 2-5) followed by a very strong increase at 6 and 9 days for WT (lanes 6 and 8 respectively). In contrast, Pt mutant showed a declining course after 3 days where the level of VP1 expression dropped to undetectable levels at 6 days and 9 days (lanes 7 and 9 respectively) similar to the pattern of early gene expression described above (Figure 1B, upper panel). Collectively, all these results suggest that, in the absence of agnoprotein, JCV and SV40 protein expression (early and late) appear to be severely down-regulated.

### Replication efficiency of JCV and SV40 greatly diminished in the absence of agnoprotein

**Figure 5:**
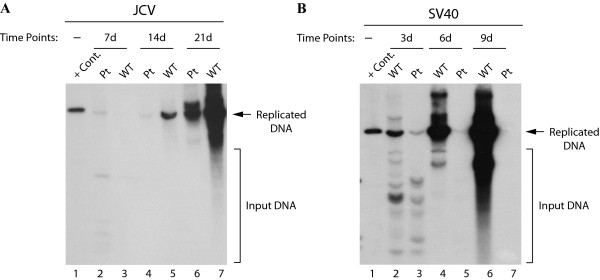
**Replication efficiency of JCV and SV40 Pt mutants**. (A) SVG-A cells were transfected with either JCV Mad-1 WT or the Pt mutant genome. Low molecular-weight DNA was isolated at the time points indicated, digested with *Bam*H I and *Dpn *I enzymes, resolved on a 0.8% agarose gel, and analyzed by Southern blotting. In lane 1, JCV Mad-1 WT genome (2 ng) digested with *Bam*H I was loaded as a positive control (+ Cont.). (B) CV-1 cells were transfected with either SV40(776) WT or its Pt mutant genome and the replication efficiency of both genomes was assessed by Southern blotting as described for panel A. Replication studies with WT and the Pt mutants for both JCV and SV40 genomes were repeated more than three times and a representative data is shown here. The input DNA (transfected), which is digested by *Dpn *I is indicated by the brackets. In lane 1, SV40(776) genome (2 ng) digested with *Bam*H I was loaded as a positive control (+ Cont.).

Since viral early and late gene expression is negatively affected in the absence of agnoprotein (Figure 1 and 4), we next assessed the functional consequences of this effect on viral DNA replication by employing a *Dpn *I replication assay. JCV Mad-1 WT or the Pt mutant genome was transfected into SVG-A cells and at 7d, 14d and 21d posttransfection, low-molecular weight DNA was isolated by the Hirt method [41]. Subsequently, newly replicated *Dpn *I-resistant DNA was analyzed by Southern blotting. It was observed that the replication efficiency of the JCV Mad-1 Pt mutant was much lower than WT (Figure 5A), even though SVGA cells constitutively express SV40 LT-Ag, which has been shown to cross-regulate JCV DNA replication [45-49]. For example, at day 14 after transfection, the level of DNA replication for Pt mutant was about 4.3-fold less than that of the WT (compare lane 4 to 5). At day 21 posttransfection, the observed difference between WT and the Pt mutant became even more pronounced (~19-fold, compare lane 6 to 7). These results are consistent with our previous findings, where we showed that replication of JCV Pt mutant was significantly hampered in the absence of agnoprotein [36]. Of note, the origin of JCV DNA replication and auxiliary DNA sequences necessary for the efficient replication of the Pt mutant remained intact after manipulations of the viral genome during the mutagenesis process, which is evident from the DNA sequencing data. Also note that SVG-A cells do not express either SV40 VP1 or SV40 agnoprotein [19] but constitutively express LT-Ag. The above findings suggest that providing LT-Ag *in trans *is not sufficient to bring the levels of JCV viral replication to that of WT in the absence of agnoprotein. In addition to SVG-A cells, we also used PHFG cells in replication assays and obtained consistent results as obtained for SVG-A cells (see additional file 1 figure S1, compare lane 6 with 7).

We next examined the replication properties of the SV40 Pt mutant compared to WT in CV-1 monkey kidney cells to see if it behaves in a similar manner observed for JCV. The results from this replication studies showed that the replication efficiency of the SV40 Pt mutant was also hampered compared to the WT (Figure 5B). The level of replicated DNA from WT virus gradually increased during the course of the infection cycle until 9th day posttransfection. However, the amount of replicated DNA for SV40 Pt mutant virus declined after 3th days following transfection reaching a point where its replication was barely detectable after day 6 of the infection cycle, which is consistent with the results obtained from viral protein expression studies in Figure 1B. The quantitative analysis of the results showed that there was approximately 120-fold more replicated DNA for the WT compared to the mutant at day 9 posttransfection.

### Stable expression of agnoprotein *in trans *restores the replication activity of the JCV Pt mutant virus

**Figure 6:**
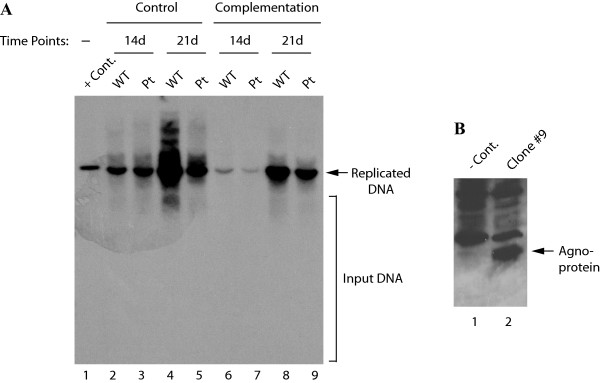
**Analysis of the replication efficiency of JCV WT and its Pt mutant in agnoprotein positive cells**. (A) SVG-A cells were stably transfected with an agnoprotein expression plasmid and clones selected as described in Materials and Methods. Cells expressing agnoprotein or the control cells (stably transfected with empty vector) were transfected with either JCV Mad-1 WT or its Pt mutant genome as indicated. Low molecular weight DNA was isolated and analyzed by *Dpn *I-Southern blot analysis as described in Figure. 5A. In lane 1, JCV Mad-1 WT genome (2 ng) digested with *Bam*H I was loaded as a positive control (+ Cont.). (B) Western blot analysis of the stable expression of agnoprotein in SVG-A cells. In lane 1, whole cell extracts prepared from the SVG-A cells stably transfected with empty vector were loaded as negative control (- Cont.). In lane 2, whole cell extract prepared from an agnoprotein positive clone was loaded.

Having established the functional significance of agnoprotein in viral replication, we asked whether the stable expression of agnoprotein *in trans *can complement the loss of agnoprotein expression from the viral background and restore the replication activity of Pt mutant to that of the WT levels. To address this question, SVG-A cells were stably transfected with an agnoprotein expression plasmid and stable clones expressing JCV agnoprotein were selected. An agnoprotein positive clone was then transfected either with JCV Mad-1 WT or Pt mutant viral genome and replicated DNA analyzed by Southern blotting. In parallel, an SVG-A control clone which was stably transfected with an empty vector was also transfected either with WT genome or Pt mutant genome. As shown in Figure 6A, a clear difference of about 11.6-fold was observed between the replication efficiency of WT and that of the Pt mutant genomes at day 21 in the control cells (compare lane 4 to 5). However, the difference between the replication efficiency of WT and that of the Pt was much lower (about 1.6-fold) when the viral genomes were separately transfected into an agnoprotein-expressing clone (compare lane 8 to 9). These data indicate that stable expression of agnoprotein *in trans *restores the replication activity of the mutant genome. It should be noted here however that there appears to be variation between the data points between the control and the complementation group. For instance, when compared the replication efficiency of the control lanes at 14^th ^day posttransfection with that of the complementation group in this figure, the latter is lower. This is most likely due to the clonal variation as well as the growth differences between the cells, since one clone is constitutively expressing agnoprotein and the other is not. We have found previously that agnoprotein markedly inhibits cell growth [16]. (This may account for this variation with respect to the level of replication of either JCV WT or the Pt mutant in control group (Agno-negative) at 14d posttransfection compared to those in the complementation group (Agno-positive) for the same day. However, the most important point in this figure is that the gap between the levels of replication of WT versus the Pt mutant gets wider as the replication cycle proceeds for the control group, where no agnoprotein was produced by the cells. For instance, while there is an approximately 12-fold difference between WT and the Pt mutant at 21d posttransfection (lanes 4 and 5), this difference decreased to ~1.6 fold for the same day data point in the complementation group (lanes 8 and 9), suggesting that providing agnoprotein *in trans *complements its loss during the replication cycle of JCV. Figure 6B demonstrates the expression level of agnoprotein in clone #9 by Western blotting.

### Analysis of the virions released from SVG-A cells transfected/infected with JCV Pt mutant

**Figure 7:**
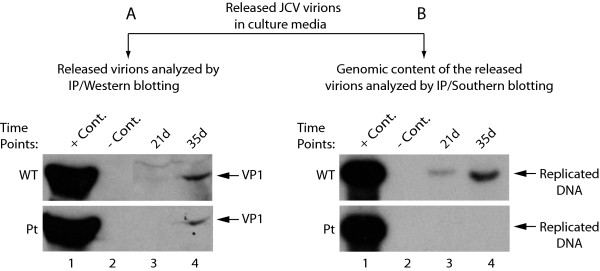
**Analysis of the virions released from PHFG cells transfected/infected with either JCV Mad-1 WT or its Pt mutant**. Supernatants from the transfected/infected PHFG cells were collected at indicated time points centrifuged at 16,000 × g to clear the cell debris and were subjected to immunoprecipitation using an anti-VP1 antibody (PAb597) to precipitate the released virions. Immunoprecipitants were divided into two equal portions, one of which was analyzed by Western blotting to detect viral capsid protein VP1 (A) and the other was analyzed by Southern blotting for the detection of the encapsidated viral DNA (B). In lane 1 in panel A, supernatant from infected cells was immunoprecipitated with anti-VP1 antibody and loaded as a positive control (+ Cont.). In lane 2 in panel A, supernatant from uninfected cells was immunoprecipitated with anti-VP1 antibody and loaded as a negative control (- Cont.). In lane 1 in panel B, JCV Mad-1 genome (2 ng) digested with *Bam*H I was loaded as a positive control (+ Cont.). In lane 2 in panel B, supernatant from uninfected cells was immunoprecipitated, digested with *Bam*H I and loaded as a negative (- Cont.).

So far, our results showed that the propagation of JCV was significantly hampered in the absence of agnoprotein. We next wanted to address the following questions: (i) is this due to possible defects occurring in the release of the virions from the infected cells or (ii) is some other mechanism involved such as the release of defective virions during the course of viral infection? To address these possibilities, we employed a virion release assay, where the released virions present in the supernatant of the transfected cells were immunoprecipitated with antibody raised against the capsid protein VP1 and samples were divided into two equal portions, one portion of which was analyzed by Western blotting (Figure 7B) and the other portion was analyzed by Southern blotting (Figure 7B). Western blot analysis of the released capsid proteins (VP1) for both WT and Pt mutant showed that virions were efficiently released from infected cells and were readily detectable at 35d posttransfection for both WT and the Pt mutant. However, analysis of the genomic content of the released virions by Southern blotting surprisingly showed that the virions released from the cells transfected with Pt mutant infected cells were mostly devoid of viral DNA compared to WT (Figure 7B). These findings suggest that viral virions are efficiently released from the infected cells in the absence of agnoprotein, and yet they are deficient with respect to the genomic content, which may account for, at least in part, why the Pt mutant virus is defective in viral propagation. The lack of detection of viral genome in released virions may be attributed to the possible defects in virion biogenesis and/or viral DNA replication processes of Pt mutant.

### Analysis of the virions released from CV-1 cells transfected/infected with SV40 Pt mutant

**Figure 8:**
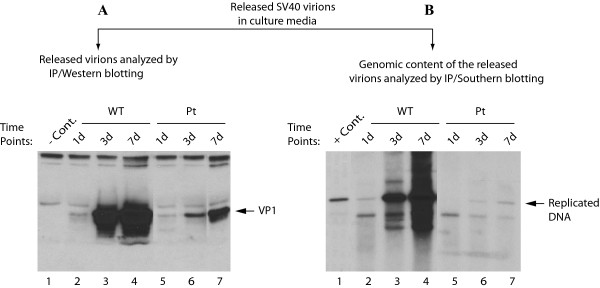
**Analysis of the virions released from CV-1 cells transfected/infected with either SV40(776) WT or its Pt mutant**. CV-1 cells were transfected with either SV40(776) WT or Pt mutant genome and supernatants from infected cells were collected at time points indicated for analysis of the released virions by Western blot for VP1 (A) and Southern blotting (B) as described for the figure legends for Figure. 7A. and Figure. 8B respectively. In lane 1 in panel A, supernatant from uninfected cells was immunoprecipitated with anti-VP1 antibody and loaded as a negative control (- Cont.). In lane 1 in panel B, SV40(776) genome (2 ng) digested with *Bam*H I was loaded as a positive control (+ Cont.).

In parallel, we also performed a virion release analysis for SV40. CV-1 cells were either transfected/infected with SV40(776) WT or its Pt mutant genome and supernatants from transfected/infected cells were collected at different time points as indicated and subjected to immunoprecipitation with anti-VP1 antibody. The immunoprecipitated samples were divided into two equal half portions. One half was analyzed for detection of viral capsid protein VP1 by Western blot and the other half was analyzed by Southern blotting to evaluate the level of viral DNA encapsidated into the released virions. As shown in Figure 8A, similar to JCV, the SV40 viral particles were efficiently released from the infected cells in the absence of agnoprotein, which was evident from the significant level of detection of VP1 for Pt mutant compared to WT (Figure 8A). However the DNA content of the released virions for Pt mutant was almost undetectable while the DNA content of WT DNA was much higher than that of the Pt mutant (Figure 8B, compare lanes 2-4 to 5-7). As for JCV, this may, at least in part, explain why SV40 agnoprotein Pt mutant cannot efficiently propagate compared to WT. Of note, there appears to be differences when compared the data for the WT and the Pt VP1 samples in Figure 8 with those obtained for Figure 4B. This apparent discrepancy can be explained as follows. These two experimental settings are different. In Figure 4B, the level of expression of VP1 was analyzed using extracts prepared from infected cells at the indicated data points. However, in Figure 8A, VP1 levels were determined in immunoprecipitated virions that are released into and accumulated in the culture media from the day of transfection on and up to the day of data collection. Due to this difference in experimental settings, variations in results are expected. However, overall, the virion release assays have demonstrated that both JCV and SV40 agnoprotein Pt mutants behave similarly with respect to the efficiency of the release of the virions from the infected cells and the deficiency of DNA content in released viral particles.

### Analysis of growth properties of SV40 Pt mutant in Cos-7 cells, constitutively expressing SV40 large T antigen

**Figure 9:**
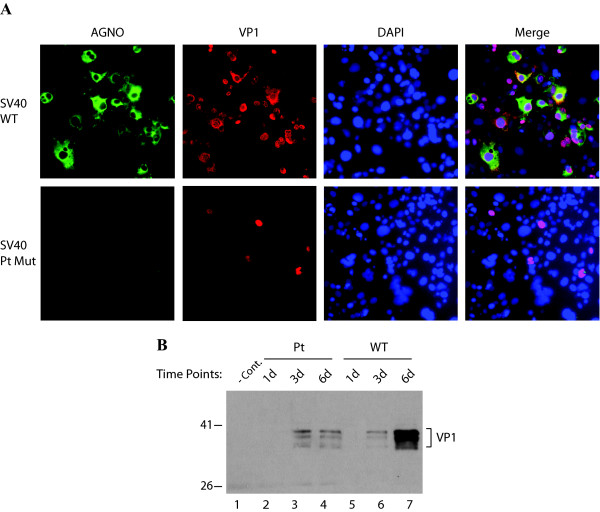
**Analysis of the growth rate of SV40 Pt mutant in Cos-7 cells by immunocytochemistry and Western blotting**. (A) Cos-7 cells were transfected/infected with either SV40(776) WT or its Pt mutant as described in materials and methods and at 6^th ^day posttransfection, cells were fixed in cold acetone, washed with 1 × PBS and incubated with both anti-agno polyclonal rabbit [19] and anti-VP1 (PAb597) monoclonal mouse primary antibodies (1:200 dilution each). After washing the primary antibodies with 1 × PBS, cells were incubated with secondary antibodies, goat anti-rabbit FITC-conjugated (green), and goat anti-mouse Rhodamine-conjugated (red) antibodies. Cells were finally washed with 1 × PBS, mounted in mounting media containing DAPI stain (Vector laboratories Inc. CA) and examined under fluorescent microscope. (B) Western blot analysis of nuclear extracts for VP1 expression, prepared from Cos-7 cells transfected/infected with either SV40 (776) WT or its Pt mutant as described for panel A above. Nuclear extracts (10 μg/each sample) were resolved on a 10% SDS-PAGE, transferred onto a nitrocellulose membrane and probed with anti-VP1 monoclonal antibody (PAb597). In lane 1, nuclear extracts from untransfected Cos-7 cells were loaded as a negative control (- Cont.).

The main regulatory protein of human (JCV and BKV) and primate (SV40) polyomaviruses is the LT-Ag, which is responsible for initiation of the viral DNA replication and transactivation of the viral late genes. We have demonstrated in this work that, in the absence of agnoprotein, these viruses are unable to replicate as efficiently as WT. Our data from replication assays in SVG-A cells (Figure 5A) showed that providing LT-Ag to the replication system in vivo, in the absence of agnoprotein, is not sufficient to alleviate the replication level of JCV Mad-1 Pt mutant closer to that of JCV Mad-1 WT. Of note, SVG-A cells constitutively express SV40 LT-Ag [40]. In order to address the same question for SV40 Pt mutant, that is whether constitutive expression of SV40 LT-Ag is sufficient to bring the replication level of SV40 Pt mutant closer to that of SV40 WT, Cos-7 cells which constitutively express SV40 LT-Ag, were transfected with either SV40 (776) WT or its Pt mutant as described in the legend for Figure 9, and the efficiency of infection was examined by both immunocytochemistry (Figure 9A) and Western blotting (Figure 9B). Overall immunocytochemistry studies showed that majority of the cells were labeled with anti-agno antibody (green) and anti-VP1 antibody (red) VP1, as expected, when cells were transfected/infected with SV40 WT (Figure 9A). However, a few of the cells (1-5%) were labeled when the cells were transfected/infected with SV40 Pt mutant (Figure 9A lower panels). In parallel studies, comparison of VP1 expression of WT with that of Pt mutant by Western blot analysis was found to be correlated with those from immunocytochemistry studies respectively (Figure 9B). Taken together, as it was observed for JCV Pt mutant replication in SVG-A cells (Figure 5A), the constitutive expression of SV40 LT-Ag in Cos-7 cells is not sufficient to alleviate the Pt replication level to that of SV40 WT. In other words, agnoprotein is required for the efficient replication of both JCV and SV40.

## Discussion

The regulatory roles of agnoprotein in JCV life cycle are not fully understood. In this work, we specifically investigated the regulatory role(s) of JCV and SV40 agnoprotein in virion release utilizing agnoprotein point mutants of each virus in which the ATG initiation codon has been removed so as to ablate expression. We found that both JCV and SV40 viral gene expression and replication were drastically reduced in the absence of agnoprotein suggesting that both viruses require agnoprotein for the successful completion of their lytic cycle. Our findings are also supported by a recent report by Myhre *et al*, in which it was demonstrated that a non-functional BKV mutant with deletions in the agnogene can be complemented in *trans *from a co-existing BKV rr-NCCR variant [38].

In addition, consistent with our findings from gene regulation studies, JCV and SV40 agnoprotein Pt mutants showed an enormous negative effect on viral DNA replication. SVG-A cells used in replication assays were derived from PHFG cells by stably transfecting a replication defective SV40 genome [40]. This cell line expresses the viral early regulatory protein, LT-Ag, but not the viral late genes including agnoprotein. Preparation of PHFG cells is labor intensive, costly, and there is variability between the batches of cell preparations, greatly affecting consistency of the results. SVG-A cells constitutively express SV40 LT-Ag, which was previously shown to cross-regulate JCV replication [45-48], but our data presented in this work convincingly showed that expression of SV40 LT-Ag alone is not sufficient for the efficient replication of neither JCV Pt mutant in SVG-A cells (Figure 5A) nor SV40 Pt mutant in Cos-7 cells (Figure 9), which also constitutively express SV40 LT-Ag. It should be noted here that the origin of DNA replication of JCV and SV40 in agnoprotein Pt mutants was intact, which was verified by sequencing. As such, the relatively low level of replication of JCV and SV40 Pt mutants compared to their WTs cannot be attributed to the unintended mutations that occurred during the mutagenesis process, which greatly affect the replication of each mutant. Therefore, it was expected that SV40 LT-Ag would up-regulate JCV replication in agnoprotein Pt mutant background regardless of the absence of agnoprotein. However, we found that agnoprotein is required for efficient regulation of JCV and SV40 replication in the context of the whole viral genome, i.e., the constitutive expression of SV40 LT-Ag is not sufficient to complement the absence of agnoprotein for the efficient replication of JCV and SV40. In fact, the results from the complementation assays support this conclusion and demonstrate that the level of replication of the JCV agnoprotein Pt mutant is alleviated to a level comparable to that of WT when agnoprotein is provided to the replication system in *trans *(Figure 6A).

We also investigated the question of whether agnoprotein has a role in the release of viral particles and found that JCV and SV40 virions are efficiently released from the infected cell in the absence agnoprotein, but they are mostly deficient in viral DNA content (Figure 7B and 8B). Our findings are in contrast with the recent report by Suzuki et al. [39] which suggests that agnoprotein functions as a viroporin and therefore may play a role in release of virions from JCV-infected cells. Our results do not support this idea since we found that viral particles were efficiently released from infected cells to the culture medium in the absence of agnoprotein. However, the released virions are not as infectious particles as those of WT due to the lack of viral encapsidated DNA, i.e., they appear to be mostly composed of empty capsids and therefore unable to initiate an efficient next round of the infection cycle. This may be the major reason why the level of propagation of agnoprotein Pt mutant is profoundly reduced compared to that of WT.

## Conclusions

The small regulatory proteins of many viruses play important roles in regulation of the viral life cycle and are therefore critical to the fine tuning of many aspects of virus-host interactions. Our findings in this report indicate that the agnoprotein of JCV and SV40 is required for efficient regulation of viral DNA replication and gene regulation. This may have functional consequences for the successful completion of the JCV lytic cycle. In light of our findings, we propose that agnoprotein may substantially contribute to the process of proportional production of viral capsid proteins versus that of viral DNA, thereby leading to the formation of a high number of optimally infectious viral particles for the next round of the infection cycle. Finally, given the fact that JCV is the etiologic agent of PML and may be involved in the induction of some of the human malignancies, such findings make agnoprotein an attractive target for therapeutic approaches to control JCV-induced diseases in the affected individuals. Understanding the molecular mechanisms associated with the regulatory functions of agnoprotein is important for unraveling the molecular secrets of the unique biology of JCV and JCV-associated diseases.

## List of abbreviations

JCV: JC virus; BKV: BK virus; SV40: Simian vacuolating virus 40; PML: Progressive multifocal encephalopathy; FITC: fluorescein isothiocyanate; PBS: Phosphate buffered saline; DMEM: Dulbecco's modified eagle's medium; LT-Ag: large T antigen, Pt: point mutant; HBSS: Hanks balanced salt solution; PHFG: Primary human fetal glial cells; FBS: Fetal bovine serum.

## Author details

Department of Neuroscience, Laboratory of Molecular Neurovirology, Temple University School of Medicine, 3500 N. Broad Street, Philadelphia PA 19140 USA

## Competing interests

The authors declare that they have no competing interests.

## Authors' contributions

MS involved in designing and implementing all the experiments; and writing the manuscript. IKS took part in implementation of all the experiments. SS made great contribution to writing and editing the manuscript. MW participated in data interpretation, writing and editing the manuscript. All authors read and approved the final manuscript.

## Supplementary Material

Additional file 1 **Analysis of the growth properties of JCV Mad-1 WT and its Pt mutant in PHFG cells**. PHFG cells were transfected/infected with either JCV Mad-1 WT or its Pt mutant genome. Low molecular-weight DNA was isolated at the time points indicated, digested with *Bam*H I and *Dpn *I enzymes, resolved on a 0.8% agarose gel, and analyzed by Southern blotting. In lane 1, JCV Mad-1 WT genome (2 ng) digested with *Bam*H I was loaded as a positive control (+ Cont.). The input DNA (transfected), which is digested by *Dpn *I is indicated by brackets. Click here for file
