# Supplementary figures and images for: Infection by agnoprotein-negative mutants of polyomavirus JC and SV40 results in the release of virions that are mostly deficient in DNA content
Source: Virol J. 2011 May 24;8:255. doi: 10.1186/1743-422X-8-255 (PMC3127838; doi:10.1186/1743-422X-8-255)

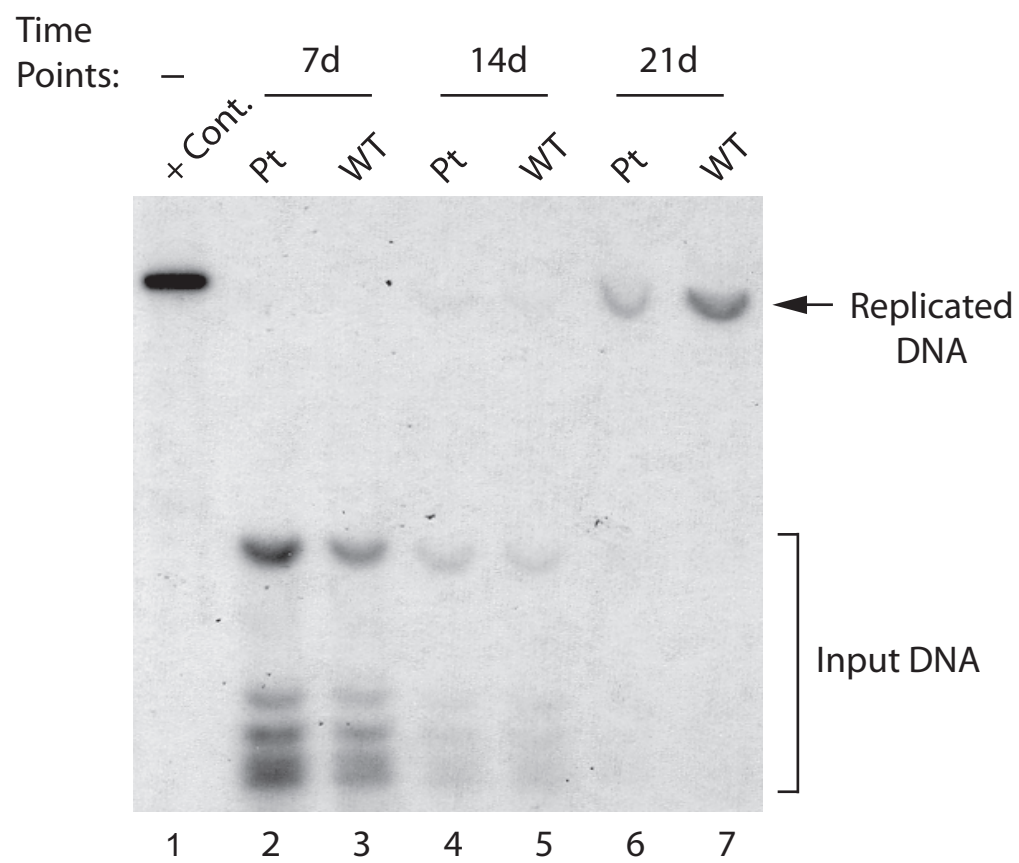

Supplement: Additional file 1 — Analysis of the growth properties of JCV Mad-1 WT and its Pt mutant in PHFG cells. PHFG cells were transfected/infected with either JCV Mad-1 WT or its Pt mutant genome. Low molecular-weight DNA was isolated at the time points indicated, digested with BamH I and Dpn I enzymes, resolved on a 0.8% agarose gel, and analyzed by Southern blotting. In lane 1, JCV Mad-1 WT genome (2 ng) digested with BamH I was loaded as a positive control (+ Cont.). The input DNA (transfected), which is digested by Dpn I is indicated by brackets. [file 1743-422X-8-255-S1.PDF]
